# Supplementary material for: Projection of premature mortality from noncommunicable diseases for 2025: a model based study from Hunan Province, China, 1990–2016
Source: PeerJ. 2020 Nov 3;8:e10298. doi: 10.7717/peerj.10298 (PMC7646306; doi:10.7717/peerj.10298)
Supplement: Supplemental Information 1 — Abbreviations: APC, Annual percent change; AAPC, Average Annual percent change; NCDs, non-communicable diseases; CVD, cardiovascular disease. a It was calculated by first converting AAPC to predicted single year change, then exponentiating to the number of study years minus one to produce overall change and its magnitude, which was finally converted to a percent change. * Statistically significant at the alpha=0.05 level. [file peerj-08-10298-s001.docx]

Table S1 Temporal trends in premature mortality from NCDs during 1990-2016, Hunan Province, China

| Diseases | AAPC | Overall change (%)^a^ | Trend1 | |  | Trend2 | |  | Trend3 | |  | Trend4 | |
| --- | --- | --- | --- | --- | --- | --- | --- | --- | --- | --- | --- | --- | --- |
|  |  |  | Years | APC |  | Years | APC |  | Years | APC |  | Years | APC |
| **Both** | | | | | | | | | | | | | |
| Total | -1.8* | -37.5 | 90~02 | -1.2* |  | 02~09 | -3.4* |  | 09~16 | -1.1* |  |  |  |
| Cancer | -0.9* | -20.1 | 90~02 | -0.3* |  | 02~09 | -2.0* |  | 09~16 | -0.7* |  |  |  |
| CVD | -2.1* | -41.6 | 90~03 | -1.4* |  | 03~08 | -4.4* |  | 08~16 | -1.5* |  |  |  |
| Diabetes | -0.2 | -5.8 | 90~01 | 2.5* |  | 01~11 | -3.0* |  | 11~14 | 1.8 |  | 14~16 | -3.7 |
| Chronic respiratory disease | -5.4* | -76.4 | 90~95 | -2.1* |  | 95~01 | -4.7* |  | 01~09 | -9.2* |  | 09~16 | -3.9* |
| Other | -1.7* | -36.1 | 90~02 | -1.3* |  | 02~09 | -3.2* |  | 09~14 | -0.4* |  | 14~16 | -1.9* |
| **Male** | | | | | | | | | | | | | |
| Total | -1.3* | -28.7 | 90~02 | -1.0* |  | 02~08 | -3.0* |  | 08~16 | -0.4* |  |  |  |
| Cancer | -0.6* | -13.4 | 90~02 | -0.3* |  | 02~08 | -1.5* |  | 08~14 | 0.2 |  | 14~16 | -1.4 |
| CVD | -1.3* | -29.2 | 90~02 | -0.9* |  | 02~08 | -3.3* |  | 08~16 | -0.5* |  |  |  |
| Diabetes | 0.5* | 15.0 | 90~01 | 2.2* |  | 01~08 | -2.4* |  | 08~14 | 1.8* |  | 14~16 | -2.1 |
| Chronic respiratory disease | -4.6* | -70.8 | 90~95 | -2.1* |  | 95~01 | -4.2* |  | 01~09 | -8.7* |  | 09~16 | -2.0* |
| Other | -1.5* | -32.1 | 90~01 | -1.4* |  | 01~09 | -2.6* |  | 09~14 | 0.4 |  | 14~16 | -1.9 |
| **Female** | | | | | | | | | | | | | |
| Total | -2.7* | -50.4 | 90~02 | -1.6* |  | 02~05 | -4.0* |  | 05~10 | -4.8* |  | 10~16 | -2.3* |
| Cancer | -1.6* | -33.6 | 90~95 | -0.9* |  | 95~02 | 0.0 |  | 02~11 | -3.1* |  | 11~16 | -1.6* |
| CVD | -3.2* | -57.5 | 90~96 | -2.6* |  | 96~02 | -1.7* |  | 02~10 | -5.0* |  | 10~16 | -3.1* |
| Diabetes | -0.9* | -20.1 | 90~01 | 2.4* |  | 01~05 | -2.2* |  | 05~09 | -5.9* |  | 09~16 | -2.2* |
| Chronic respiratory disease | -6.9* | -84.2 | 90~96 | -2.9* |  | 96~02 | -6.0* |  | 02~11 | -10.7* |  | 11~16 | -5.5* |
| Other | -2.1* | -41.6 | 90~98 | -1.4* |  | 98~02 | -0.4 |  | 02~10 | -4.0* |  | 10~16 | -1.5* |

**Abbreviations**: APC = Annual percent change; AAPC = Average Annual percent change; NCDs = non-communicable diseases; CVD = cardiovascular disease.

^a^ It was calculated by first converting AAPC to predicted single year change, then exponentiating to the number of study years minus one to produce overall change and its magnitude, which was finally converted to a percent change.

* Statistically significant at the alpha=0.05 level.
