# Supplementary material for: Projection of premature mortality from noncommunicable diseases for 2025: a model based study from Hunan Province, China, 1990–2016
Source: PeerJ. 2020 Nov 3;8:e10298. doi: 10.7717/peerj.10298 (PMC7646306; doi:10.7717/peerj.10298)
Supplement: Supplemental Information 2 — The standardized population, the extracted death data from the GBD study 2016, and the corresponding variables in the death data. [file peerj-08-10298-s002.zip › Supplementary 2/Varible note-NCDs 90-16.docx]

Definition of variables from Table S2:

Location_id=Location (504=Hunan);
Year_id=Year; Sex_id=Sex (1=male, 2=female, 3=both);
Age_group_id=Age_group( yearsold) 11=30-, 12=35-,13=40-, ... , 18=65- ;
CAUSE_ID=Causes of death 409=total NCDs, 410=Cancer, 491=CVD, 508=Chronic respiratory diseases, 587=Diabetes;
Num_mean=Number of death; rt_mean=Mortality rate; pop=population.
